# A paired emitter–detector diode-based photometer for the determination of sodium hypochlorite adulteration in milk

**DOI:** 10.1038/s41598-023-33527-y

**Published:** 2023-04-17

**Authors:** Narges Bastan, Mazaher Ahmadi, Tayyebeh Madrakian, Abbas Afkhami, Sina Khalili, Mohsen Majidi, Mohammadreza Moradi

**Affiliations:** 1 Faculty of Chemistry, Bu-Ali Sina University, Hamedan, 6517838695 Iran; 2 Research Center for Molecular Medicine, Hamadan University of Medical Sciences, Hamadan, Iran

**Keywords:** Analytical chemistry, Nutrition

## Abstract

This paper reports on developing a low cost but efficient paired emitter–detector diode (PEDD)-based photometer. The photometer consists of a white light-emitting diode (LED) as the emitter diode, an RGB LED as the detector diode, and a multimeter for recoding the signal. The developed PEDD-based photometer was utilized for the determination of liquid bleach adulteration in cow milk samples. *N,N*-Diethyl-*p*-phenylenediamine sulfate aqueous solution of pH 6 was used as a probe to monitor the presence of residual active chlorine in milk. The results showed that the developed method could be used to determine sodium hypochlorite in the concentration range of 0.5 to 20.0 ppm Cl_2_ with 0.14 and 0.46 ppm Cl_2_ limit of detection and limit of quantification, respectively. The intraday and interday precisions of the method at two concentration levels of 5.5 and 13.7 ppm Cl_2_ were 1.04% and 0.52%, and 1.81% and 1.02%, respectively. The recoveries of 114.2% and 106.9% were obtained for 5.5 and 13.7 ppm Cl_2_ concentrations levels, respectively. Real sample analyzes results showed that “maybe” liquid bleach adulteration in milk is the case for local distributors of raw milk.

## Introduction

As a highly complex food of nutritional importance, cow milk contains proteins, lactose, and fats^1^. Milk's short shelf life has always been a problem. Although some special treatment during obtaining, storage, and transportation of milk can assure its quality, still its shelf life is limited and should be stabilized chemically or physically. The heat treatment followed by rapid cooling for pasteurization of raw milk is widely used for increasing shelf life and reducing the health risk of milk^2^. However, some small businesses milk producers utilize various adulterations to increase the shelf life at a low cost, potentially risking the end-users health^3–7^. These adulterations may include simple dilution with water, dilution with lower quality milk, and addition of chemicals for increasing the shelf life such as hypochlorite, formaldehyde, hydrogen peroxide, dichromate, or increasing the final product yield such as melamine, starch, urea, surfactants, and oils^8–10^.

Because milk is such a sensitive product, natural deterioration is the fundamental concern that bleach solutions address^8^. As a result of the bleach solution's contact with the milk components, chemical reactions occur, neutralizing the medium and eventually lowering microbial growth, preventing the emergence of odor, taste, and viscosity changes^9,11^. Proteins and lipids are likely to be the molecular groups most affected by oxidation in milk. The harmful effects of consuming oxidized proteins and lipids have been thoroughly studied in the literature, particularly the rise in systemic oxidative stress, and the formation of reactive oxygen species in animals^12,13^. In 2018, a research using mass spectrum data analysis discovered five oxidized milk components following sodium hypochlorite adulteration including (*E*)-2-((9-hydroxy-10-oxotetradecanoyl)oxy)-3-(phosphonooxy)propyl tetradec-9-enoate, d-galacturonic acid calcium salt, d-glyceraldehyde-3-phosphate/(3-hydroxy-2-oxo-propoxy) phosphonic acid/(2*S*)-2-phospholactate, and (*E*)-2-((5-oxopent-3-enoyl)oxy)-3-(palmitoyloxy)propyl(2-(tri-methylammoniom) ethyl) phosphate)^8^. These compounds are the result of the hypochlorite adulterant's oxidative properties that influences milk composition even at low concentrations, most likely by oxidizing lipid double bonds and producing stable lipid alcohols. Milk is a good source of fat-soluble vitamins and lipids^14^. Furthermore, these components contribute to the distinctive flavor of dairy products and milk, as well as their physical properties. While oxidized forms may have a reduction or absence of these properties^15^ or, in the worst-case scenario, alter biochemical functions leading to deleterious effects, these oxidized endproducts could act as harmful chemicals that activate inflammatory responses in the circulatory system as well as organs such as the kidney, liver, gut, and lung^16^.

Therefore, the determination of bleach adulteration in milk is critically important. However, most conventional methods for the determination of residual chlorine have failed to address this issue since bleach solution reacts with milk compounds. A study utilized the Rupp test^17^ for the determination of residual chlorine in milk after the addition of hypochlorite and found that after the addition of 50 ppm active chlorine to a milk sample, no residual chlorine was found after only 5 min at room temperature using the utilized method^18^. Another study utilized iodometric titration for the determination of the kinetics of the reaction of sodium hypochlorite with milk. This study showed that the main components of milk that react rapidly with sodium hypochlorite were lactoglobulin, lactalbumin, casein, lactose, insoluble lipoprotein, soluble lipoprotein, and fat globule membrane lipids^19^. A recent study in Iran showed that the government-recommended methods for the determination of bleach solution adulteration in milk were not applicable for this aim^20^, which may be due to the fast reaction of hypochlorite anions with milk and low residual concentration of it in milk received by the end-users. Another study in Brazil indicated the limitations of the legally described tests in detecting the fraudulent addition of preservatives and neutralizers, besides difficulties in carrying out these tests; they may not be able to assure the absence of these substances in milk^21^.

In 1962, visible light-emitting diodes (LEDs) with p-n junctions producing a narrow band of light wavelengths were discovered^22,23^. Because of their compact size, robustness, low cost, and high efficiency, LEDs have found widespread application in the miniaturization of optical sensors. Miniature spectroscopic instruments have decreased in size due to development in liquid crystal displays (LCDs), fiber-optics, laser diodes, computing, etc.^24,25^. The light source is an important part of portable instruments, and the growth of laser technology has enabled the development of miniature gratings to reduce the size of spectrometers. This is done by using tungsten-halogen and fluorescent lighting as broadband sources, laser, LCDs, and LEDs as narrow-band sources, and by dispersion of optical filters to increase the sensitivity of measurements and reduce spectral interferences^26,27^. The development of handheld spectrometers depends on the improvement of detector technologies. An ideal detector is one that responds in a wide wavelength range with high sensitivity and speed, has a linear response range and low noise, reduces sample consumption, and allows miniaturization^28^. To achieve this, photomultiplier tubes (PMTs), photodiode detector array (PDA), charge-coupled device (CCD), complementary metal-oxide semi-conductors (CMOS), and paired emitter detector diode (PEDD) have been developed^25,29^. Miniaturized spectrophotometers suffer from low detection sensitivity due to limited optical path length^30^, so approaches such as multireflection cells^31^, microfluidic systems, axial-direction^32^, liquid-core waveguides^33^, confined drop-based systems, and cavity ring-down spectroscopy^34^ have been developed to improve sensitivity. Smartphones are mobile devices that combine computing and communication features, making them easier to use at the point of care. They are used to design biosensors and bioelectronics, and are equipped with a high-resolution camera with the capability to store, analyze, and transfer images. White LED flash as a light source and CMOS array as a photodetector are used, while diffraction grating as a monochromator can be attached to the camera to design a smartphone-based spectrometer^35^. The camera can distinguish various colors based on RGB filters, although cannot distinguish specific wavelengths. An attaching accessory such as transmission or reflective diffraction gratings is used to disperse entry radiation before detection using the camera of the smartphone as a detector^36^. It should be noted that the response from different smartphone are not comparative due to different resolutions of built-in cameras. The development of universal smartphone photometers/spectrophotometers is of interest^37,38^.

LEDs are used as the light source in these miniaturized spectrophotometers. However, LEDs may be utilized as both the detector and light source the in optical sensors using paired emitter–detector diodes (PEDD)^23,39^. PEDD-based photometers have a low fabrication cost, a low power consumption, are easily miniaturized, and have a good signal-to-noise ratio response across a wide wavelength range^23^. In addition, their output is a direct pulse-duration-modulated signal, which eliminates the need for an expensive analog-to-digital conversion^40^. Because of these benefits, PEDD-based optical sensors are now used in a variety of miniature photometers, as well as a flow-through optical sensor for flow analysis and chromatography^41–43^. Commercially available photometers/colorimeters are either single or multi-channels. In most of them, LEDs are used as the light sources not the detector. Single channel photometers utilize a LED of particular emission wavelength for a specific use. On the other hand, multi-channel photometers utilize optical filters for each channel. Thus, these instruments have been designed to detect only one analyte (or a variety of analytes of the same absorbance characteristics)^29,44^.

Therefore, the main aim of this study is to develop an easy setup photometer of low cost, enabling end-users of the milk cycle to ensure that no bleach solution alteration has been done on their dairy milk product. To this end, we have developed a PEDD-based photometer and provided the reagent necessary to monitor residual hypochlorite in milk. The developed PEDD-based photometric system utilizes a natural white LED as the emitter diode and a RGB LED chip (wavelengths: 460–465 nm for blue, 520–525 nm for green, and 620–625 nm for red LEDs) as the detector diode. The voltage of each detector LED was measured using a multimeter. Tri-color LED could be useful as three absorption values can be recorded simultaneously, then using the obtained signals (i.e., R, G, and B) or ratio of these values for multi analyte analysis. N, N-Diethyl-p-phenylenediamine sulfate (DPD) aqueous solution of pH 6 was used as a probe to monitor the presence of residual active chlorine in milk.

## Results and discussion

### Calibration curve in water

**Figure 1:**
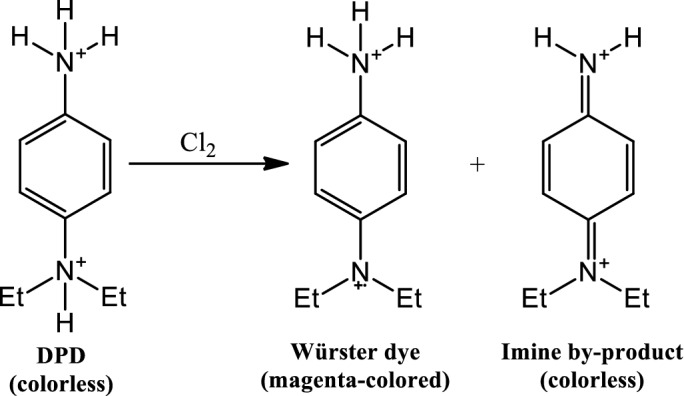
Mechanism and products of DPD-chlorine reaction.

**Figure 2:**
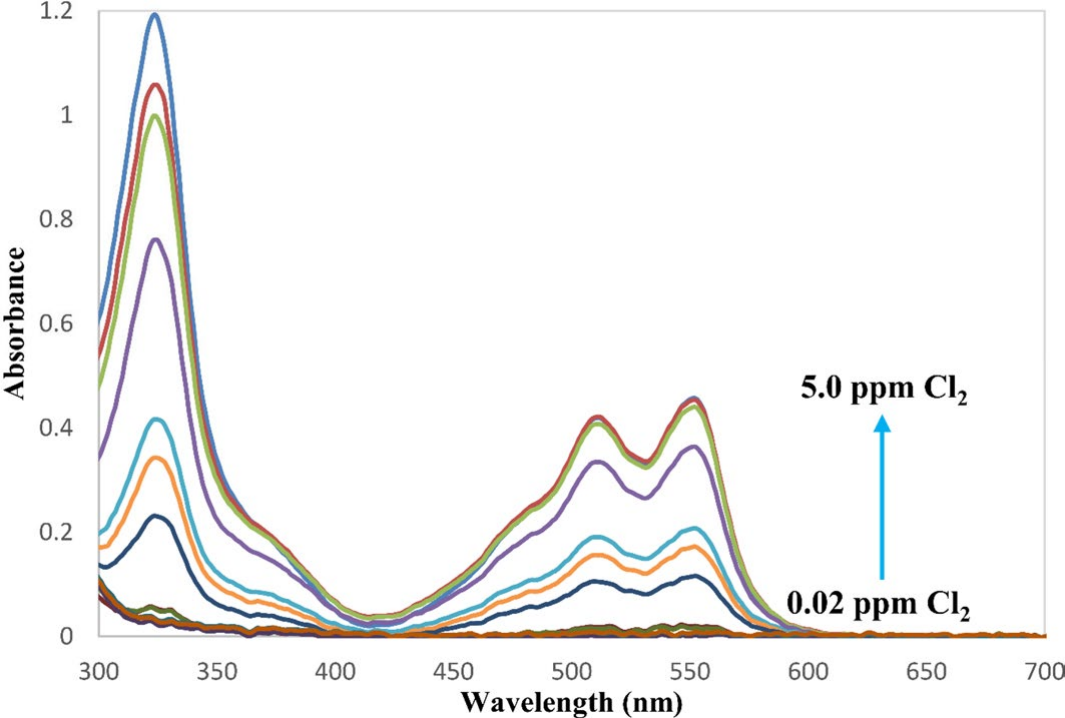
The calibration spectra obtained using the benchtop spectrophotometer for the determination of residual chlorine in water (conditions: 100 μL DPD reagent and 100 μL buffer solution of pH 6 were added to a total volume of 5.0 mL).

The DPD method involving the oxidation of the DPD reagent of pH 6 by sodium hypochlorite was repurposed to monitor sodium hypochlorite adulteration in milk^45–47^ (Fig. 1). To this end, the method was first validated in water using the developed PEDD-based photometer against the benchtop spectrophotometer by adding 100 μL DPD reagent and 100 μL buffer solution of pH 6 to the standard working solutions of 0.02 to 5 ppm Cl_2_. The calibration curves were obtained using three diode detectors of the developed photometer as well as the benchtop spectrophotometer at 326, 513, and 553 nm. Figure 2 and Table 1 show the obtained results. As shown in Fig. 2, three maximum absorbance wavelengths (i.e., 326, 513, and 553 nm) can be considered for the construction of calibration curves. Among them, the 326 nm wavelength provides the highest sensitivity (Table 1). However, for the development of the PEDD-based photometer, a white emitter LED was utilized that does not emit wavelengths in the ultraviolet range. Therefore, to compare both systems, the visible range must be considered. The linear range and the slope of the calibration curves obtained by the developed photometer, using the green diode detector, were wider than the benchtop spectrophotometer at both studied wavelengths. For the PEDD-based photometer, the green diode detector results in the highest sensitivity and widest linear range. The green diode detector is sensitive to wavelengths lower than 520 nm corresponding to adsorption of the solution at 512 nm in accordance with the complementary colors concept (a red-colored solution absorbs the green spectrum of the light source)^48^. Therefore, it makes sense that the green detector results in the highest sensitivity. For the red diode, the saturation of the detector can occur due to a broader range of wavelengths that the detector is sensitive to, which leads to a lower sensitivity.

**Table 1:** A summary of the calibration curve equations and linear ranges obtained using the benchtop spectrophotometer and the developed PEDD-based photometer (conditions: 100 μL DPD reagent and 100 μL buffer solution of pH 6 were added to a total volume of 5.0 mL).

Instrument	Calibration equations	Linear ranges (ppm Cl_2_)	R^2^
Benchtop spectrophotometer	A (326 nm) = 0.3178 C (ppm Cl_2_) + 0.0723	0.1 – 3.0	0.9867
	A (513 nm) = 0.1316 C (ppm Cl_2_) + 0.0399	0.1 – 3.0	0.9651
	A (553 nm) = 0.1417 C (ppm Cl_2_) + 0.0452	0.1 – 3.0	0.9632
PEDD-based photometer	^a^A (G detector) = 0.014 C (ppm Cl_2_) + 0.0801	0.75 – 5.0	0.9938
	^b^A (G detector) = 0.6743 C (ppm Cl_2_) − 0.0184	0.02 – 0.1	0.9881
	A (B detector) = 0.0159 C (ppm Cl_2_) + 0.0087	0.1 – 5.0	0.9857
	^a^A (R detector) = 0.0104 C (ppm Cl_2_) + 0.0196	0.5 – 5.0	0.9898
	^b^A (R detector) = 0.2703 C (ppm Cl_2_) − 0.0087	0.03 – 0.1	0.9736

For the PEDD-based photometer, the applied voltage was 2.5 V.

^a^Higher concentration range.

^b^Lower concentration range.

### Optimization of the PEDD-based photometric system

DPD-1 method was originally developed for the determination of free active chlorine water. There is no report on the repurposing of this method for milk matrix since milk matrix is very complex compared to water. Benchtop photometers and spectrophotometers utilize low-energy lamp sources that are designed for the analysis of transparent or semi-transparent matrixes. Therefore, these instruments cannot be utilized for recording the small changes in the absorbance of the matrix due to the formation of the DPD oxidized pink product in the presence of sodium hypochlorite. On the other hand, high matrix dilution cannot be an option since it decreases the limit of quantification. Therefore, there is a need for the development of a system that can be accompanied by a high-intensity source beam. Another problem in the determination of liquid bleach adulteration in milk is the fact that the components of milk react rapidly with sodium hypochlorite and leave ultra-trace free active chlorine available^18^. This makes the method calibration very difficult. Therefore, it is important to develop the method calibration procedure first.

#### Method calibration procedure

The effect of incubation time on the reaction of sodium hypochlorite with milk component was studied using the developed PEDD-based photometer. To this end, the green diode was utilized as the detector; the applied voltage to the emitter LED was 2.8 V, and a 1 cm quartz standard cuvette was used. 500 µL buffer solution of pH 6 and sodium hypochlorite solution were added to 2.5 mL milk samples. Then, the solutions were diluted to the total volume of 5.0 mL with deionized water to make a theoretical 100 ppm Cl_2_ initial concentration. 400 µL portions of the DPD reagent were added to these solutions at different incubation times.

The absorbance of the solution decreases as the incubation time increase due to the fast reaction of hypochlorite anions with milk components. Therefore, it is necessary to develop a procedure for standard sample preparation when photometric/colorimetric determination of sodium hypochlorite in milk is intended. In this study, the standard solutions were prepared by the initial reaction of sodium hypochlorite solution and DPD regent at pH 6 in the absence of the milk matrix. After the reaction completeness, ca. 2 min, the milk samples were added and the mixtures were diluted up to the desired volume. For the method validation and spiked/recovery experiments, the same procedure was used.

Another important issue with the DPD reagent is the reagent reaction with dissolved oxygen under light illumination. To confirm the effect of dissolved oxygen, high-purity nitrogen was slowly purged into the reaction vessel containing deionized water (flow rate ~ 150 mL min^−1^) for 10 min. Then, the DPD reagent was added and its UV–vis absorbance spectra after placing the solution in the PEDD-based photometer (applied voltage of 3.2 V) for 5 min was recorded using the benchtop spectrophotometer. The results showed that nitrogen-purged DPD solution was stable but DPD undergoes oxidation in the oxygen containing DPD solution. Although this would not be the case when benchtop photometers and spectrophotometers are used, this study showed that DPD reagent reaction could lead to false-positive results using the developed PEDD-based photometer when low dilution ratios of milk samples were used. Therefore, the milk samples were diluted by half with deionized water to avoid the need for high-applied voltage to the emitter LED. In addition, the signal reading was fast, *ca*. 1 min, to avoid light-catalyzed DPD oxidation.

#### Type of the detector LED

Previous research on the development of PEDD-based photometers have used rational paired LED systems to increase system selectivity, as the name implies^43,49–51^. The emitter LED's wavelength should be shorter than the detector LED's. For the first time, an RGB detector LED and a white emitter LED are used in this study for the determination of sodium hypochlorite adulteration in milk. This method resulted in the creation of a multipurpose photometer that can be utilized for purposes other than the primary goal of this study.

To choose the best detector LED that provides both selective and sensitive determination of the oxidized DPD pink product, calibration curves in the concentration range of 0.5 to 20.0 ppm Cl_2_ using each detector LED were constructed individually under the same conditions. The results are shown in Table 2. As can be seen, the blue detector does not provide an analytically acceptable calibration curve. The green and red detector LEDs provide comparable efficiencies, but saturation of the red detector can occur due to a broader range of wavelengths that the detector is sensitive to. Therefore, to obtain a wider linear range and a higher selectivity, the green detector was utilized for the rest of the study.

**Table 2:** A summary of the calibration curve equations obtained using the developed PEDD-based photometer in the concentration range of 0.5 to 20.0 ppm Cl_2_ using different detector LEDs (conditions: 200 μL DPD reagent and 500 μL buffer solution of pH 6 were added to appropriate sodium hypochlorite solution portions).

Detector LED	Emission wavelength (nm)	Detection wavelength (nm)	Calibration equations	R^2^
Green LED	520–525	< 520	A_G_ = 0.0263 C (ppm Cl_2_) + 0.0321	0.9858
Red LED	620–625	< 620	A_R_ = 0.0262 C (ppm Cl_2_) + 0.0307	0.9683
Blue LED	460–465	< 460	A_B_ = 0.0173 C (ppm Cl_2_) + 0.0643	0.7597

Then, 2.5 mL milk samples were added to the mixture and the mixtures were diluted to the final volume of 5.0 mL using deionized water. For the PEDD-based photometer, the applied voltage was 2.8 V.

#### The power supply voltage

**Figure 3:**
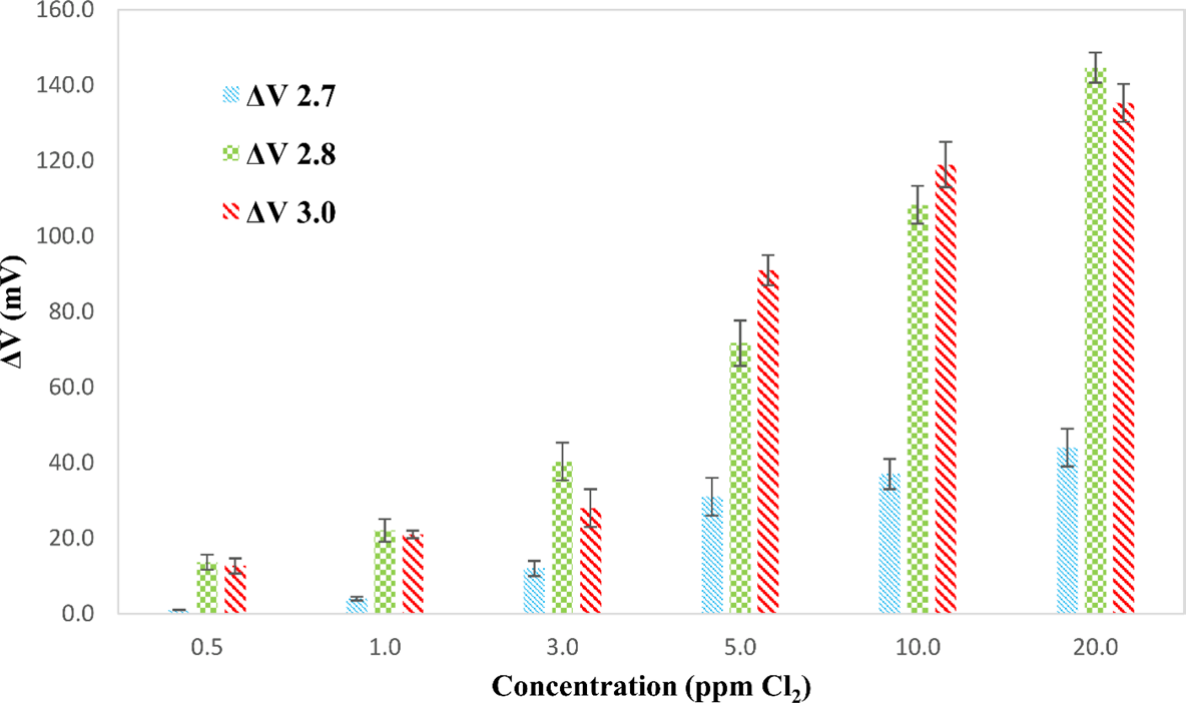
Effect of the applied voltage on the recorded green detector LED response for different concentrations of sodium hypochlorite in milk using the developed PEDD-based photometer (conditions: 500 µL buffer solution of pH 6, sodium hypochlorite solution, and 400 µL portions of the DPD reagent were mixed. Then, 2.5 mL milk samples were added to the solutions and the solutions were diluted to the total volume of 5.0 mL with deionized water to make theoretical 0.5 to 20.0 ppm Cl_2_ initial concentrations).

The voltage provided to the detector LED is an essential parameter. It functions as a two-edged sword. The saturation of the detector and DPD oxidation by dissolved oxygen become increasingly important as the applied voltage increases. However, as it lowers, so does the sensitivity. For investigation of the effect of applied voltage, 200 μL DPD reagent and 500 μL buffer solution of pH 6 were added to appropriate sodium hypochlorite solution portions. Then, 2.5 mL milk samples were added to the mixture and the mixtures were diluted to the final volume of 5.0 mL using deionized water to make final concentrations of sodium hypochlorite in the range of 0.5 to 20 ppm Cl_2_. The change of the voltage of the green detector was calculated by subtracting the voltage of the detector in the presence and absence of the DPD reagent at three applied voltages (i.e., 2.7, 2.8, and 3.0 V). The findings (Fig. 3) demonstrated that raising the applied voltage from 2.7 to 2.8 V enhanced the detector response for all concentrations tested. However, raising the applied voltage to 3.0 V resulted in a reduction in detector performance at low concentrations. As a result, the applied voltage of 2.8 V was chosen as the best value.

### Method validation

The developed method was validated in terms of accuracy, sensitivity, and precision. To this end, 500 µL buffer solution of pH 6, sodium hypochlorite solution, and 400 µL portions of the DPD reagent were mixed. Then, 2.5 mL milk samples were added to the solutions and the solutions were diluted to the total volume of 5.0 mL with deionized water to make 0.5 to 20.0 ppm Cl_2_ initial concentrations. The green LED was used as the detector in the PEDD-based photometer. The voltage supplied to the emitter LED was 2.8 V. Under the aforementioned conditions, the calibration curve was created with three replicates for each concentration. The calibration curve equation was A_G_ = 0.0263 C (ppm Cl_2_) + 0.0321 (R^2^ = 0.9858) for the concentration range of 0.5 to 20.0 ppm Cl_2_. The limit of detection (LOD) and limit of quantification (LOQ) were calculated as 3S_b_/m and 10S_b_/m, respectively, where S_b_ is the standard deviation of the blank signal (n = 7) and m is the slope of the calibration curve according to IUPAC recommendation^52^. The LOD and LOQ were 0.14 and 0.46 ppm Cl_2_, respectively. The intraday and interday (three consecutive days) precisions of the method were evaluated at two concentration levels of 5.5 and 13.7 ppm Cl_2_. The intraday precisions, calculated as the coefficient of variation, were 1.04% and 0.52% for 5.5 and 13.7 ppm Cl_2_ concentrations, respectively (n = 3). The interday precisions were 1.81% and 1.02% for 5.5 and 13.7 ppm Cl_2_ concentrations, respectively. The spiked/recovery experiments at two concentration levels of 5.5 and 13.7 ppm Cl_2_ was performed to evaluate the accuracy of the developed method. The recoveries of 114.2% and 106.9% were obtained for 5.5 and 13.7 ppm Cl_2_ concentrations levels, respectively.

### Real samples analysis

To evaluate the applicability of the developed method, seven raw cow milk samples from local distributors in Hamedan, Iran, were collected in the morning. The samples were analyzed using the developed method for the determination of sodium hypochlorite under the optimized experimental conditions. The result (Table 3) showed that two of seven analyzed milk samples were suspected of liquid bleach adulteration. Also, the samples were analyzed using the iodometric titration reference method^53^. The results showed that the iodometric titration was unable to detect the residual amount of sodium hypochlorite due to its low sensitivity. To investigate the accuracy of results, raw milk 6 and raw milk 7 were spiked at two concentration levels of 2.0 and 5.0 ppm Cl_2_ after adding DPD reagent to the samples. The recoveries obtained using the developed method were between 95.0%-105.0% (Table 4) showing acceptable method accuracy. Furthermore, the spiked samples were analyzed using the iodometric titration reference method. The F-test results showed that there is no significant difference between the variances of the two methods (the precision of the two methods) at the 95% confidence level. The accuracy of the method was also evaluated against the iodometric titration reference method. The t-test results showed that there is no significant difference between the means of the two methods at the 95% confidence level.

**Table 3:** Determination of liquid bleach adulteration in milk samples using the developed method (n = 3).

Sample	Residual free chlorine concentration (ppm Cl_2_)	
	Developed method	Iodometric titration
Raw milk 1	ND^ɑ^	ND
Raw milk 2	ND	ND
Raw milk 3	ND	ND
Raw milk 4	ND	ND
Raw milk 5	ND	ND
Raw milk 6	0.7 (2.45%)^b^	ND
Raw milk 7	1.7 (2.68%)	ND

^ɑ^Not detected.

^b^Coefficient of variation.

**Table 4:** The results of the spiked/recovery experiments conducted for the validation of the developed method for the determination of liquid bleach adulteration in milk (n = 3).

Sample	Spiked (ppm Cl_2_)	Developed method		Iodometric titration		F-test^b^	t-test^b^
		Found (ppm Cl_2_)	Recovery (%)	Found (ppm Cl_2_)	Recovery (%)		
Raw milk 6	–	0.7 (2.45)^ɑ^	–	ND	–	–	–
	2.0	2.8 (2.32)	105.0	2.9 (3.21)	110.0	2.1	1.079
	5.0	5.6 (1.86)	98.0	5.7 (2.42)	98.0	1.7	0.709
Raw milk 6	–	1.7 (2.68)	–	ND	–	–	–
	2.0	3.6 (1.95)	95.0	3.7 (2.83)	100.0	2.2	0.972
	5.0	6.8 (1.42)	102.0	6.9 (2.34)	104.0	2.8	0.651

^ɑ^Coefficient of variation.

^b^F_critical_ at 95% confidence level (ʋ_1_ = ʋ_2_ = 2) = 19.0, t_critical_ at 95% confidence level (ʋ = 4) = 2.776.

## Conclusions

This study contributes to the idea of "analytical chemistry beyond laboratory walls" by reporting on the development of a low-cost yet efficient PEDD-based photometer. Photometers are rarely found in regular people's homes these days since they are both costly and difficult to operate. However, the produced photometer has the potential to be portable, and its low-cost and simple technology might be expanded to typical consumers' houses. To boost the device's usability, the photometer must be supplemented by an inbuilt potentiometer and a mobile phone app. Nonetheless, the analytical chemist should calibrate the equipment.

## Materials and methods

### Reagents and materials

*N,N*-Diethyl-*p*-phenylenediamine sulfate (DPD) salt (purity ≥ 98.0%) was purchased from Sigma-Aldrich Company (St. Louis, Missouri, United States). Aqueous solutions of DPD reagent of 1100 mg L^−1^ concentration were prepared in deionized water (resistivity ≥ 18.2 MΩ) weekly and stored in dark condition at a fridge. The solutions were purged with nitrogen gas before storage. Sodium hypochlorite aqueous solution (6–14% active chlorine) was purchased from Supelco, Inc. (Bellefonte, Pennsylvania, United States). It was standardized using the iodometric titration method (ISO 7393-3:1990). Sodium hypochlorite working solutions of 100 and 500 ppm active chlorine (ppm Cl_2_) were prepared from the standardized solution daily in deionized water. Phosphate buffer of pH 6 (0.5 M) was prepared using KH_2_PO_4_ and Na_2_HPO_4_, both analytical grades, purchased from Sigma-Aldrich.

### Apparatus

To illuminate the LED emitter diode, a MEGATEK power supply type MP-3003S (Albania) was employed. The detector LED voltage was measured using a SOAR digital multimeter model ESC820D-L (Utah, United States). For the spectrophotometric analysis of the result of the hypochlorite and DPD reaction in water, a WPA UV/Vis spectrophotometer model Lightwave II was utilized. The PEDD-based photometer was built using a PERSIAN3D FDM 3D printer model Founder 2X (Iran).

### Milk samples pretreatment

Cow milk samples were obtained daily from trusted farmers in the morning. The samples were collected in sterilized polyethylene bottles (steam sterilization). Seven different samples were mixed in the same dilution ratios and were used for the construction of the calibration curves daily. For the real sample analysis, different raw milk samples were collected from the local dairy stores. All the samples were stored in a fridge and were brought to room temperature before analysis.

### Paired emitter–detector diode-based photometer

**Figure 4:**
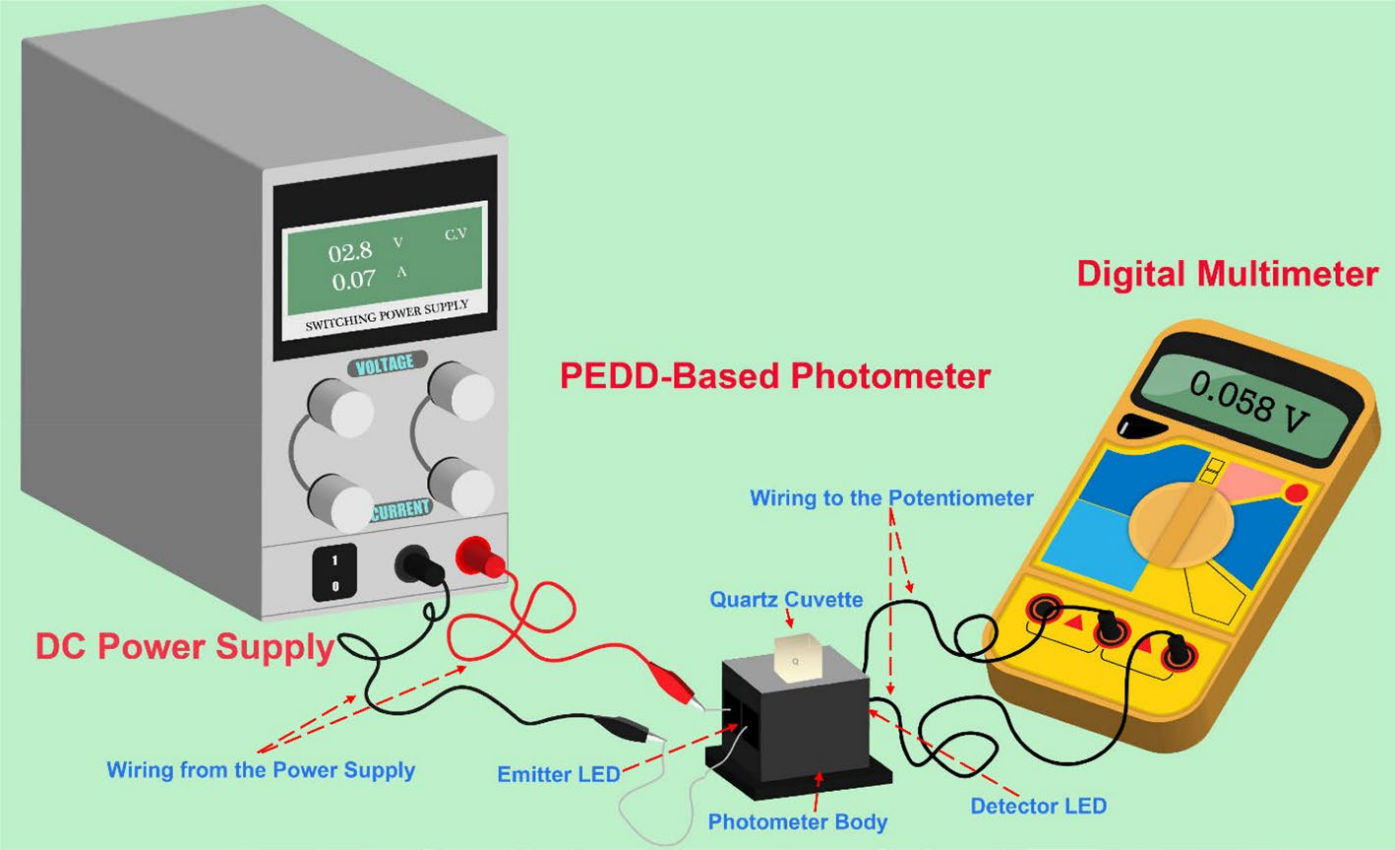
A schematic illustration of the developed PEDD-based photometric system for the determination of residual active chlorine in milk samples.

Figure 4 depicts a schematic representation of the developed PEDD-based photometric method for determining residual active chlorine in milk samples. An FDM 3D printer was used to create a rectangular cube photometer body with dimensions of 3 × 2 × 3 cm and a cubic hole (11.1 × 1.1 × 2.8 cm) in the center for inserting a cuvette. The emitter diode was a 3W natural white Bridgelux chip LED. The power supply was used to power it. The detector was a 6-pin 3W YD-XGJH RGB SMD LED chip (wavelengths: 460–465 nm for blue, 520–525 nm for green, and 620–625 nm for red LEDs). The voltage of each detector LED was measured using a multimeter. A quartz cell with a light path of 1 cm was employed.

### Photometric method for residual active chlorine determination in milk

500 µL buffer solution of pH 6 and 400 µL portions of the DPD reagent were mixed. Then, 2.5 mL milk samples were added to the solutions. The solutions were diluted to the total volume of 5.0 mL with deionized water. The green LED was used as the detector in the PEDD-based photometer. The voltage supplied to the emitter LED was 2.8 V. The voltages of the detector LED before and after the addition of DPD reagent, V_0_ and V, were recorded and used to compute solution absorbance using the equation according the Beer–Lambert Law (Absorbance = ɛ × b × C = log (I_0_/I)), where I_0_ refers to the blank signal intensity (represented as V_0_ in this manuscript) and I refer to the sample signal intensity (represented as V in this manuscript)^54^:

1 $$Absorbance=log\left(\frac{{V}_{0}}{V}\right).$$

## Data Availability

Experimental data will be available on request, please contact the corresponding author at m.ahmadi@basu.ac.ir.
